# Syria as a War-Torn Country: How Prepared Are We for the Next Pandemic?

**DOI:** 10.1055/s-0045-1814441

**Published:** 2026-02-18

**Authors:** Bassam Hassan

**Affiliations:** 1 Department of Cardiology, Tishreen University Hospital, Tishreen University (Latakia University), Latakia, Syria

In late 2021, a young physician at a public hospital in Damascus had to choose which of two critically ill coronavirus disease 2019 (COVID-19) patients would receive the last available intensive care unit (ICU) bed. There were no ventilators left, and oxygen supplies were running dangerously low. This scenario, emblematic of Syria's overwhelmed health care system, illustrates how COVID-19 did not create new problems—it magnified those left by years of conflict, underfunding, and migration of health professionals.

## Syria's Struggle Amid the COVID-19 Crisis

More than a decade of conflict has decimated Syria's health infrastructure. By 2019, nearly half of public hospitals and primary health care centers were partially or completely nonfunctional, largely due to damage or staff shortages. 1 When COVID-19 arrived, Syria was ill-equipped to respond, and the global crisis served as a wake-up call—an immense challenge, but also a rare opportunity to rethink and rebuild the Syrian health care system.

Data published during the COVID-19 pandemic in Syria indicate a mortality rate reaching 100% among ICU patients who required, but were unable to access, mechanical ventilation. Furthermore, overall mortality of hospitalized patients witnessed a fivefold increase by the first 2 months following the pandemic onset in Syria, rising from 16.7% in June 2020 up to 49.6% in August, testifying to the rapid exhaustion of Syria's health care system early in the pandemic. 2

Care for COVID-19 patients in Syria was constrained by the shortage of ICU beds, ventilators, and essential medications. However, attributing these challenges to the COVID-19 pandemic would be misleading, as they stem from a health system long debilitated by war.

In most countries, efforts to control COVID-19 and similar crises have understandably focused on urgent, short-term response frameworks. However, greater emphasis should be placed on long-term planning that balances the continuity of essential health services with emergency preparedness and response. In a recent World Health Organization (WHO) analysis of COVID-19 Preparedness and Response Plans (CPRPs) for 106 countries, researchers found that among 154 studied CPRPs, less than half included maintenance of essential health care services, and only 7% contained monitoring and evaluation components. 3

## Upcoming Challenges for the Syrian Health Care System

Over more than 13 years of war, Syria has lost a significant portion of its population, with more than six million refugees displaced across the globe. 4 While about 1.3 million Syrians have sought asylum in Europe, the majority reside in low- and middle-income countries such as Lebanon, Iraq, and Egypt—many of which already struggle with underdeveloped health care systems. 5 6 This limited capacity has contributed to a growing burden of noncommunicable diseases among refugees.

In addition, more than 220,000 Syrians in Turkey live in crowded camps with inadequate sanitation, incomplete vaccination coverage, and restricted access to health care. 7 These conditions have facilitated the resurgence of communicable diseases such as tuberculosis, leishmaniasis, hepatitis A, and measles. 8 While many refugees hope to return to Syria once conditions allow, this anticipated return will likely place additional pressure on the already fragile health system. Syrian health authorities should prepare for a sudden increase in both communicable and noncommunicable disease burdens and strengthen surveillance, vaccination, and primary care services accordingly.

## Medical Research in Syria

Conducting research in Syria is essential to understanding the country's unique public health landscape and to strengthening both local capacity and global health equity. However, this process has been severely hindered by insufficient training, brain drain, and the absence of structured mentorship systems. 9

Additionally, insights drawn from direct engagement in COVID-19 research within multiple Syrian hospitals indicate critical challenges facing researchers on the ground. These include paper-based patient records, poor documentation and reporting of in-hospital patient data, and variability in reported information among hospitals.

Fortunately, international experience offers valuable models for addressing these challenges in resource-limited and conflict-affected settings. Approaches such as external collaboration, mentor bridging, and promoting a sustainable research culture have all shown promise. 10

While developing a comprehensive, data-driven research infrastructure across Syria remains a long-term goal, innovative local initiatives have already begun to emerge. Notably, a study by Alahdab et al 11 tested an evidence-based curriculum delivered online and demonstrated that such approaches are both feasible and effective in disaster-stricken areas like Syria. Building on this momentum, a recent locally developed evidence-based medicine (EBM) in Cardiology program at Latakia University integrated clinical teaching with interactive journal club sessions, enabling participants to directly apply EBM principles to real cardiology cases. The program's outcomes showed marked improvements in knowledge and confidence among medical students and residents, highlighting the potential of low-cost, context-adapted educational models to strengthen research and learning cultures in conflict-affected settings (conference presentation, data not yet published). 12

## A Note on Mental Health

The level of investment in mental health is an excellent indicator of advancement in any health care system. Indeed, there is a novel and growing subfield of psychiatry known as war or military psychiatry, focused on the mental health consequences in veterans and civilians affected by armed conflict, and the Syrian population is no exception. 13 A recent nationwide survey published in 2021 found that 44% of people inside Syria likely have a severe mental disorder and 36.5% have full symptoms of posttraumatic stress disorder. 14 Similar trends have also been noted among medical residents. 15 While, to the best of our knowledge, there has been no research on such a scale assessing the psychological burden of COVID-19 on the Syrian population, we speculate a superimposed detrimental effect of the pandemic that widened the gap in mental disorders, affecting both social and economic well-being. We encourage governmental efforts in Syria to address this gap through incorporating psychiatric and mental health care into primary care services and expanding access to psychiatric care at the community level. 16

## Looking to the Future: What Shall We Do?

During crises, resilient health systems must adapt to rapidly changing circumstances. In Syria, many challenges revealed during the pandemic were in fact longstanding weaknesses of the system.

Currently, most Syrian health managers are physicians without formal training in health administration, underscoring the need for specialized public health leadership. Positions involving health policy, disaster management, and service planning should be led by professionals with expertise in health systems management, public health, and health economics. Investing in such a workforce is essential for national recovery and preparedness.

Successful regional examples already exist. A number of neighboring countries such as Iraq and Jordan adopt the Field Epidemiology Training Program (FETP), which—incorporated into the Ministry of Health and assisted by the WHO and Centers for Disease Control and Prevention—has proven crucial in strengthening pandemic preparedness.

FETP graduates have contributed to surveillance, protocol development, data analysis, and rapid response coordination, demonstrating the effectiveness of structured, practice-based public health training. 17

To ensure readiness for future emergencies, Syria must prioritize three areas: data digitization and information systems, expansion of applied public health training such as FETP, and integration of mental health into primary care services. Without these foundational steps, the next pandemic could have even more devastating consequences.

The time to act is now. We call on Syrian health authorities, international donors, and the global academic community to invest in sustainable systems of health governance, data infrastructure, and workforce training. Syria's people—both at home and across the diaspora—have shown extraordinary resilience. With the right tools and sustained commitment, they can also lead the way in rebuilding a safer and healthier future.

## References

[1] Fouad F M Sparrow A Tarakji A Health workers and the weaponisation of health care in Syria: a preliminary inquiry for The Lancet-American University of Beirut Commission on Syria Lancet 2017 390(10111):2516 2526 28314568 10.1016/S0140-6736(17)30741-9

[2] Hanafi I Alzamel L Alnabelsi O Sallam S Almousa S Lessons learnt from the first wave of COVID-19 in Damascus, Syria: a multicentre retrospective cohort study BMJ Open 2023 13 07 e065280 10.1136/bmjopen-2022-065280 PMC10360434 37474170

[3] Mustafa S Zhang Y Zibwowa Z COVID-19 preparedness and response plans from 106 countries: a review from a health systems resilience perspective Health Policy Plan 2022 37 02 255 268 34331439 10.1093/heapol/czab089 PMC8385840

[4] Alhaffar M HDBA Janos S Public health consequences after ten years of the Syrian crisis: a literature review Global Health 2021 17 01 111 34538248 10.1186/s12992-021-00762-9 PMC8449996

[5] Yucesahin M M Sirkeci I Demographic gaps between Syrian and the European populations: what do they suggest? Border Crossing. 2017 7 02 207 230

[6] Melhem N Kreidieh K Ramia S The Syrian refugees crisis brings challenges to the health authorities in Europe: hepatitis a virus is a case in point Eur J Epidemiol 2016 31 07 711 714 27194123 10.1007/s10654-016-0163-5 PMC7088383

[7] Assi R Özger-İlhan S İlhan M N Health needs and access to health care: the case of Syrian refugees in Turkey Public Health 2019 172 146 152 31235210 10.1016/j.puhe.2019.05.004

[8] Blanchet K Fouad F M Pherali T Syrian refugees in Lebanon: the search for universal health coverage Confl Health 2016 10 12 27252775 10.1186/s13031-016-0079-4 PMC4888673

[9] Hanafi I Kheder K Sabouni R Factors influencing research productivity among Syrian medical professionals amidst conflict: a case-control study BMC Med Educ 2024 24 01 747 38992638 10.1186/s12909-024-05681-y PMC11241956

[10] El Achi N Papamichail A Rizk A A conceptual framework for capacity strengthening of health research in conflict: the case of the Middle East and North Africa region Global Health 2019 15 01 81 31779660 10.1186/s12992-019-0525-3 PMC6883714

[11] Alahdab F Alabed S Al-Moujahed A Evidence-based medicine: a persisting desire under fire Evid Based Med 2017 22 01 9 11 27965267 10.1136/ebmed-2016-110608

[12] Hassan B Evidence-Based Medicine in Cardiology: An innovative approach to integrating research and clinical learning in SyriaPresented at: Association for Medical Education in Syria (AMES) Conference2025; November 7–8, 2025; Damascus, Syria. Conference presentation; data not yet published

[13] Jain N Prasad S Czárth Z C War psychiatry: identifying and managing the neuropsychiatric consequences of armed conflicts J Prim Care Community Health 2022 13 21501319221106625 10.1177/21501319221106625 PMC9218442 35726205

[14] Kakaje A Al Zohbi R Hosam Aldeen O Makki L Alyousbashi A Alhaffar M BA Mental disorder and PTSD in Syria during wartime: a nationwide crisis BMC Psychiatry 2021 21 01 2 33388026 10.1186/s12888-020-03002-3 PMC7778805

[15] Soqia J Alameer M B Yakoub-Agha L Challenges of poor sleep quality and mental health issues among Syrian medical residents in 22 major hospitals across Syria J Sleep Res 2025 34 04 e14469 39873384 10.1111/jsr.14469

[16] Radfar A Ferreira M M Sosa J P Filip I Emergent crisis of COVID-19 pandemic: mental health challenges and opportunities Front Psychiatry 2021 12 631008 34349675 10.3389/fpsyt.2021.631008 PMC8326372

[17] Al Nsour M Bashier H Al Serouri A The role of the Global Health Development/Eastern Mediterranean Public Health Network and the Eastern Mediterranean Field Epidemiology Training Programs in preparedness for COVID-19 JMIR Public Health Surveill 2020 6 01 e18503 32217506 10.2196/18503 PMC7104707

